# A Mini-Review on Recent Developments in Anti-Icing Methods

**DOI:** 10.3390/polym13234149

**Published:** 2021-11-27

**Authors:** Adelya Kenzhebayeva, Baglan Bakbolat, Fail Sultanov, Chingis Daulbayev, Zulkhair Mansurov

**Affiliations:** 1 Institute of Combustion Problems, Almaty 050012, Kazakhstan; aakenzhik@gmail.com (A.K.); fail_23@bk.ru (F.S.); chingis.daulbayev@yandex.ru (C.D.); zmansurov@kaznu.kz (Z.M.); 2 Baikonurov Mining and Metallurgical Institute, Satbayev Kazakh National Technical University, Almaty 050013, Kazakhstan; 3 Department of Chemistry and Chemical Technology, Al-Farabi Kazakh National University, Almaty 050040, Kazakhstan; 4 National Laboratory Astana, Nazarbayev University, Nur-Sultan 010000, Kazakhstan

**Keywords:** anti-icing, hydrophobicity, heating coatings, nanomaterials

## Abstract

An aggressive impact of the formed ice on the surface of man-made objects can ultimately lead to serious consequences in their work. When icing occurs, the quality and characteristics of equipment, instruments, and building structures deteriorate, which affects the durability of their use. Delays in the adoption of measures against icing endanger the safety of air travel and road traffic. Various methods have been developed to combat de-icing, such as mechanical de-icing, the use of salts, the application of a hydrophobic coating to the surfaces, ultrasonic treatment and electric heating. In this review, we summarize the recent advances in the field of anti-icing and analyze the role of various additives and their operating mechanisms.

## 1. Introduction

Winter is the most difficult period for the operation of roads in many countries of the world. Along with relevant phenomena during this period such as snowstorms, blizzards and snowfalls, icing is a serious problem for the field of transport and communications. This creates difficulties in the movement of vehicles and in ensuring the safety of road users, and service organizations are not always able to solve this problem.

Like other abrasive de-icing products, the utilization of salts is a passive approach to de-icing roads. In terms of composition, anti-icing salts can be divided into three categories: chloride-containing, non-chloride and a mixed one. The first group includes sodium, magnesium, potassium, and calcium chlorides. Sodium chloride (NaCl) is the most popular material because of its abundance. It is traditionally used both in granular form, often mixed with sand, other salts or gravel, and in the form of a saline solution. The theoretical principle of using salts is based on lowering the freezing point, the temperature at which a substance reaches the state of coexistence of solid and liquid phases: when salt is added, the water vapor pressure will be lower than the vapor pressure of ice, and only as a result of lowering the temperature, the phases reach equilibrium [1,2,3,4]. In the case of NaCl, the freezing point drops to −21 °C [5].

**Figure 1:**
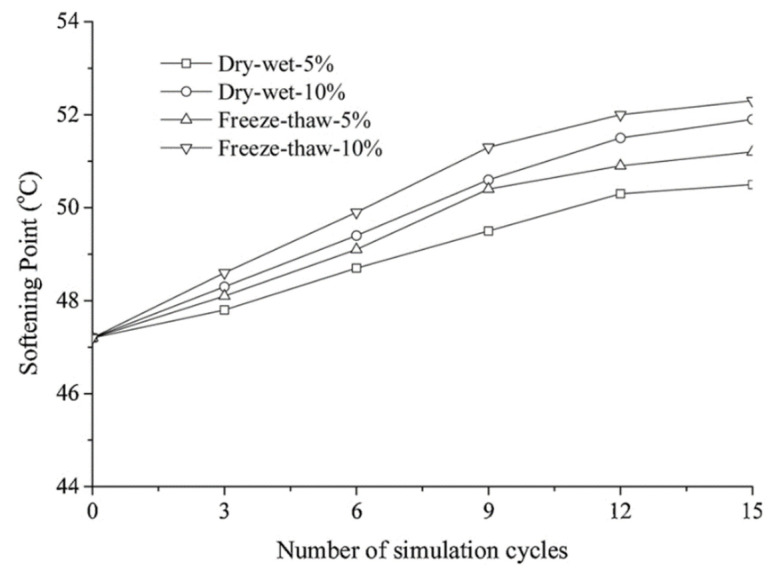
The result of the bending beam rheometer test: the dependence of the modulus of hardness of the Zhenhai#70 asphalt on the number of drying—wetting and freezing—thawing cycles. Reprinted with permission from [8].

The use of salts as an anti-icing agent is a widespread tactic that has significant disadvantages. Above all, it is accompanied by so-called salt erosion, violating the integrity of the road surface and structural parts, due to which their service life is noticeably reduced [6]. Moreover, corrosion occurs even at relatively low humidity levels due to the hygroscopic properties of salts [7]. Experimental simulations of drying–wetting and freezing–thawing cycles in [8,9,10,11,12,13,14] showed the effect of a salt medium on an asphalt pavement with an increase in the drying rate of the bitumen binder. For example, after 15 cycles of drying–moistening with 5% and 10% NaCl solutions, the modulus of hardness of the asphalt-limestone pavement increase to 20.2% and 30.3%, respectively, and after 15 freezing–thawing cycles with similar solutions—up to 28.4% and 38.2% (Figure 1). The adsorption of asphalt molecules to NaCl molecules is lower than that with binder molecules, as a result of which a decrease in the strength of the material is observed [15,16]. Peel adhesion and surface-free energy tests have demonstrated the same results [17]. According to Feng et al. [18], the damage to asphalt mixes occurs in stages. First, expansion of water reduces the indirect tensile strength, which is an indirect characteristic of strength. Thereafter, the separation layer between the asphalt and the salt-based de-icing agent layer gradually disappears with the observed decrease in the total weight.

Zhou et al., revealed a decrease in the ductility of asphalt samples from 9.2% to 27.6% with an increase in the concentration of NaCl solution to 24% at a temperature of 10 °C. This is due to the formation of large crystals by Cl- ions, which block the bond between the molecules of the asphalt, as well as an increase in the proportion of asphaltenes and resins in its composition, with a subsequent decrease in proportion of light components [1,19]. As a result, NaCl addition causes a significant performance change, aggravating a faster deterioration of the pavement.

**Figure 2:**
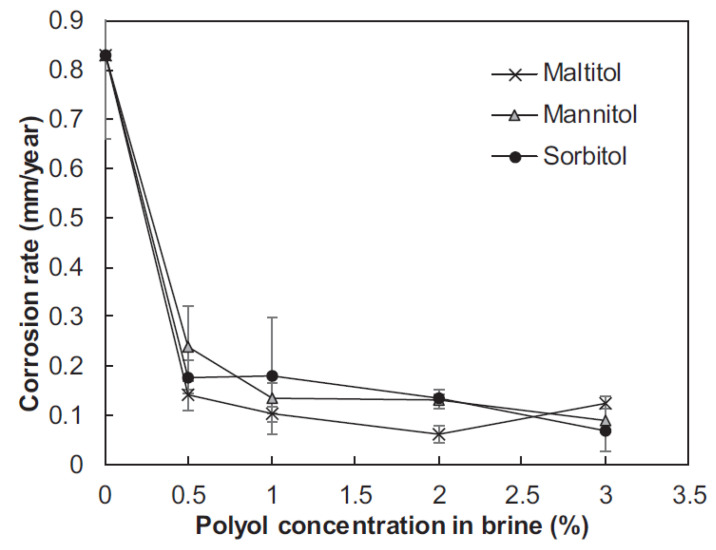
ASTM A572 corrosion rate versus polyol concentration in de-icing solution. Reprinted with permission from [7].

For a greater de-icing effect with less environmental impact, the addition of various organic substances is also proposed. Sajid et al. [7] have added polyols (maltitol, glucite and mannitol), obtained from corn, a bio-waste product, by catalytic hydrogenation, to a 23.3% NaCl saline solution in order to improve the quality of the anti-icing salt. These corn-derived polyols were added as inhibitors for the corrosion reduction incorporating environmental friendliness and low cost, and the results of the surface analysis of the obtained materials demonstrated the presence of a protective oxide layer on the steel samples. Thus, even with a glucite content of 0.5 wt.% in the solution, there was a decrease in the average corrosion rate by 79%—from 0.83 to 0.18 mm per year (Figure 2). These polyhydric alcohols contain so-called heteroatoms, which are corrosion inhibitors and active sites for adsorption on metal surfaces [20].

However, chloride-based de-icing salt remains one of the main causes of increased salt content in aquatic systems. This process is called secondary salinization and leads not only to a decrease in the amount of fresh water in the sources, but also to an excess of the threshold of chronic toxicity for freshwater organisms, negatively affecting their physiology, reproductive ability, immune functions, interactions between species, etc. [21,22,23,24,25]. Irrigation of the soil with such water poses a threat to agriculture and the entire agricultural sector, limiting plant growth and even leading to deformation of fruits due to an increase in osmotic load and the manifestation of different levels of ion-specific toxicity in different species, as well as a decrease in soil aggregation [26,27,28,29,30]. The use of chloride salts as anti-icing coatings are not diminishing, and this creates additional environmental problems.

Calcium magnesium acetate (CaMg_2_(CH_3_COO)_6_) is being considered to be a non-toxic alternative to non-degradable chloride salts to eliminate road icing problems. With a eutectic temperature of −27 °C, it is safe for the environment and is mainly used in aerodromes. The main disadvantages of using this material are its high cost (twenty times higher than that of traditional NaCl) and secondary pollution during its commercial production based on the chemical reaction of dolomite lime with acetic acid of petroleum origin [31,32,33]. Biological methods of obtaining the desired product take much longer time and, in general, have shown themselves to be ineffective. For example, as a result of the biomass fermentation process, only 4% solutions of acetic acid are obtained, while 25% solutions are usually required to obtain high-quality CaMg_2_(CH_3_COO)_6_ in the desired amount [33].

## 2. Anti-Icing Hydrophobic Compounds

**Figure 3:**
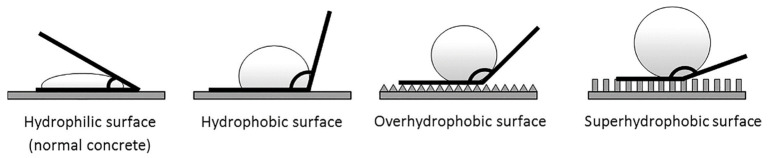
Hydrophilic (0° ≤ h ≤ 90°), hydrophobic (90° < h), very hydrophobic (120° ≤ h < 150°) and superhydrophobic (150° ≤ h ≤ 180°) surfaces, where h is the wetting angle value. Reprinted with permission from [34].

The ability of the surface to repel water, hydrophobicity, is characterized by the contact angle h (Figure 3) [34]. Depending on the angle, materials are divided into hydrophobic (90° < h), very hydrophobic (120° ≤ h < 150°) and superhydrophobic (150° ≤ h ≤ 180°) [35,36]. Application of hydrophobic de-icing coatings is a passive approach for prevention of icing. Due to their low surface energy and roughness, they are not only waterproof, but also resistant to erosion. Even with inevitable adhesion, ice can be easily removed from such surfaces due to the low adhesion force [37,38,39,40,41,42]. As a component of coatings, hydrophobic materials are extremely practical in construction in places where maintenance can be problematic (tunnels, underground parking, building foundations), and in places below groundwater level, where it is necessary to constantly ensure the dryness of water-saturated soil. After all, it is known that hydrophobic surfaces have excellent self-cleaning properties, which consequently extend the life of coatings [43].

Teflon has long been known as an effective antifriction hydrophobic material [44,45,46]. Nahipkyzy et al. found that the adhesion of a mixture of Teflon and polyphenylene sulfide used as a superhydrophobic coating on aluminum surfaces is five times greater than that of pure Teflon, making ice removal much easier. In addition, at −18 °C, ice formation on super hydrophobic surfaces took less time and did not make the structure heavier [47].

**Figure 4:**
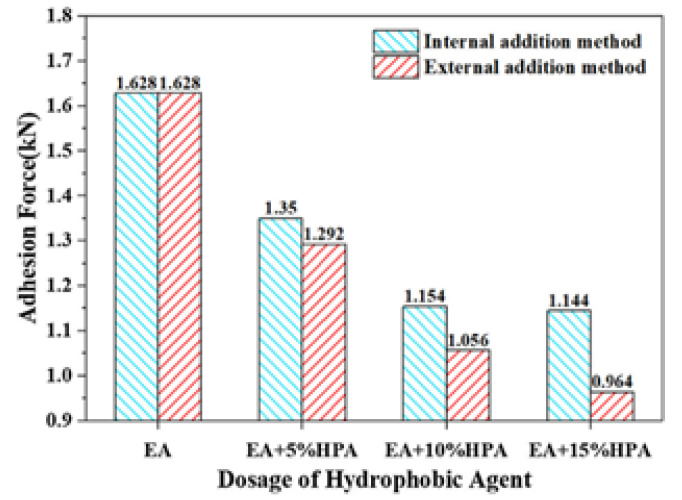
Comparison of the values for adhesion force for different methods of a hydrophobic agent addition. Reprinted with permission from [48].

Han et al. demonstrated that the synthesized hydrophobic agent (polytetrafluoroethylene powder) in emulsified asphalt reduces adhesion between the surface and the formed ice, not only depending on the amount of polytetrafluoroethylene in the material, but also on the method of its addition to the mixture. The adhesion force after 8-h freezing at a temperature of −10 °C with external addition of the agent decreased by about 40%, while for the method of internal addition it increased by 30%, resulting in weaker hydrophobicity (Figure 4). In general, ice layers on all samples of hydrophobic emulsified asphalt were fragile and layered. Water absorbed by conventional asphalt clogs the smallest holes in the porous structure. Based on this phenomenon, according to the authors, the formed ice cover is more homogeneous and uniform compared to the hydrophobic coating, where individual water droplets tend to aggregate on the surface and the ice is not only distributed locally, but also easily displaced under load [48].

**Figure 5:**
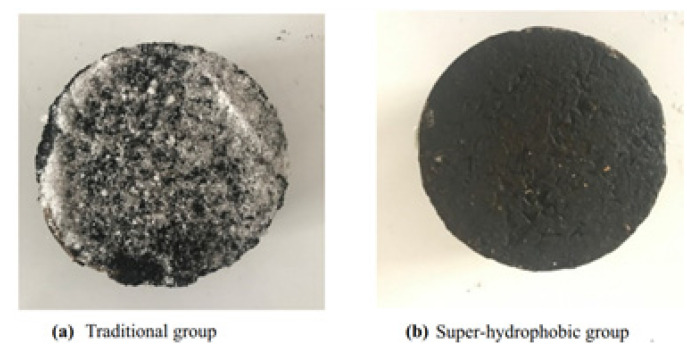
Morphology of the ice layer on the surfaces of the samples after the simulation of frosty conditions. Reprinted with permission from [49].

**Figure 6:**
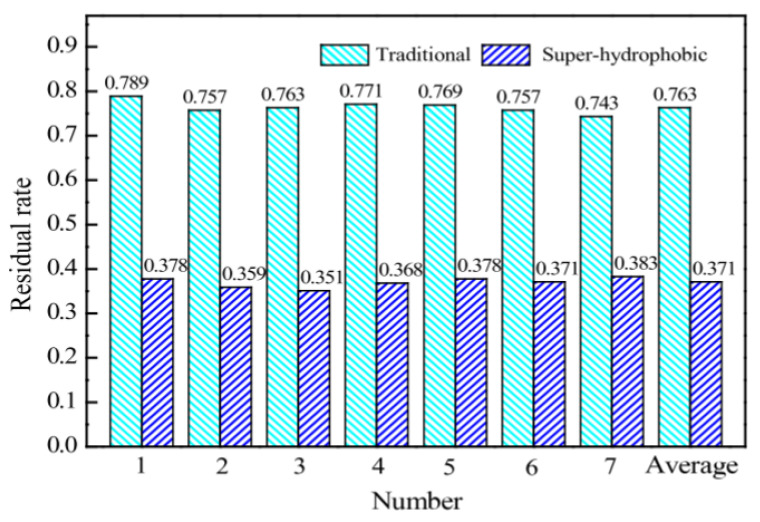
Residual ice formation rate under hail conditions. Reprinted with permission from [49].

Gao et al. studied the main properties of a superhydrophobic asphalt pavement consisting of fluorosilane and nanoparticles of silicon dioxide (SiO_2_). After artificial simulation of frosty conditions (1 h of freezing at a temperature of −10 °C and a humidity of 85%), the superhydrophobic asphalt sample remained practically dry (Figure 5), as the water droplets practically bounced off, “springing” on the surface. This is due to the point contact between a drop of water and the presence of papillary-hierarchical micro- and nanostructures of the coating, which creates surface tension. Another explanation for this phenomenon can be a slowdown in the exchange of heat between the drop and the surface due to the air layer that appears on the surface of micro- and nanostructures. Hail modeling (the formation of artificial ice with a thickness of 8 mm on samples upon freezing at a temperature of 10 °C for 2 h) also showed that the superhydrophobic coating significantly slows down the icing process during the secondary ice formation. The residual ice formation rate on this material turned out to be much lower compared to the corresponding indicator of the uncoated sample (Figure 6), which can significantly increase the road capacity [49].

**Figure 7:**
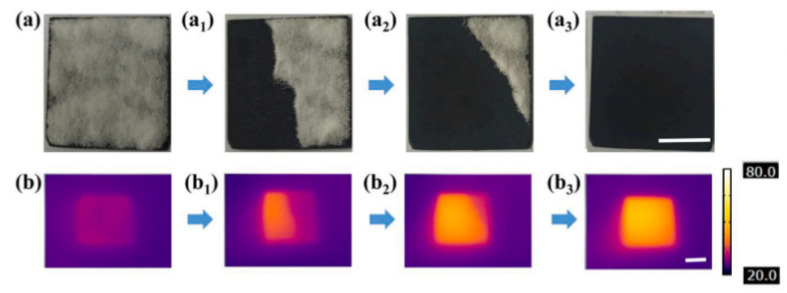
(**a**–**a_3_**) Self-cleaning test of superhydrophobic photothermal coating; (**b**–**b_3_**) thermograms of surfaces corresponding to (**a**–**a_3_**) after one irradiation process. Reprinted with permission from [43].

The hydrophobic material obtained by Xue et al., based on melanin nanoparticles fabricated from cuttlefish juice and SiO_2_ nanoparticles exhibited the ability to photothermal conversion at a temperature of −20 °C, which slowed down the icing time of water droplets on the surface [43]. Melanin in the composition of the material has a strong light-absorbing ability in a wide range, effectively converting solar energy into heat [50,51]. The use of infrared solar radiation, which is highly permeable, instead of electricity saves energy. After 72 h of irradiation with ultraviolet lamps, the material did not lose its properties. The aforementioned “air cushion” and fluorine-containing compounds serve as a kind of a barrier against erosion and corrosion, providing the chemical resistance of the coating. The self-cleaning property plays an important role in maintaining the efficiency of photothermal conversion, since the equilibrium temperature of a clean coating is much higher (Figure 7) [43].

**Figure 8:**
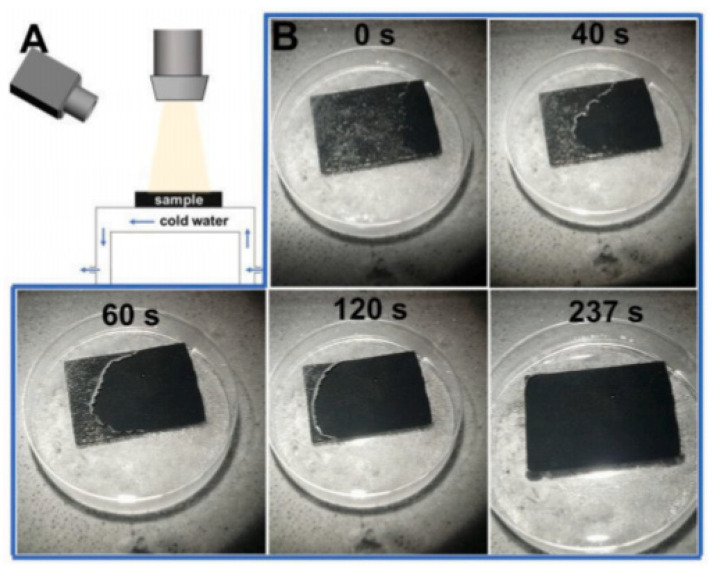
(**A**) Scheme of a photothermal ice removal device; (**B**) the process of removing ice with a thickness of about 1 mm after one irradiation (petri dish diameter is 3.2 cm). Reprinted with permission from [52].

In the report of Zheng et al., a magnetically sensitive superhydrophobic coating formed by dispersing carbonyl iron in polydimethylsiloxane followed by candle flame treatment demonstrates the strength of a two-layer fluorine-free material. Low surface energy and micro- and nano-structure are caused by polydimethylsiloxane and iron particles, respectively. The introduction of photothermal particles led to the melting of 1 mm thick ice in 237 s (Figure 8), after which the melt water also rapidly rolled off the surface due to the self-cleaning property of the resulting hydrophobic materials. During irradiation of the damaged coating with sunlight, the components of polydimethylsiloxane tend to migrate to the outer layers and, thus, restore the hydrophobicity of the material. The indisputable advantages of this coating are its magnetic response and flexibility, due to which it can be applied even on curved surfaces without the use of adhesives [52].

It is necessary to take into account the toxicity of the components contained in hydrophobic coatings for the environment during operation, as well as for the human organism during production. Among the materials mentioned above, polydimethylsiloxane can be cited as an example. Its degradation in soil is initiated with hydrolysis, accelerated by clay minerals, which are the main components of the soil. The primary product of this process is dimethylsilandiol, which can evaporate in the atmosphere and subsequently get oxidized in the presence of sunlight [53]. Regarding the SiO_2_ nanoparticles, the toxicologists believe that their effect on the human organism may have other adverse effects compared to micron-sized silica due to their small size: in vitro and in vivo studies have shown that they can cause different side effects [54,55,56,57,58].

**Figure 9:**
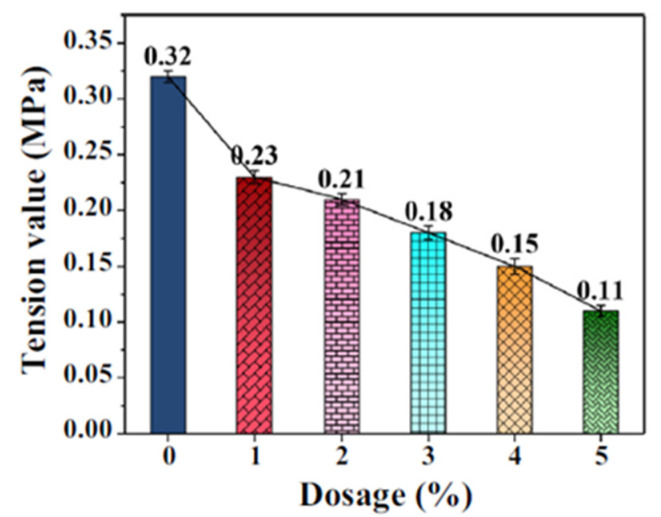
Adhesion between asphalt and ice. Reprinted with permission from [54].

A more environmentally friendly alternative was proposed by Meng et al., who prepared an emulsified asphalt coating using a biological antifreeze protein (AFP). A deep-sea cod, whose skin was the source of protein powder used in the mentioned study, and many other organisms have investigated to produce such substance in the course of evolution. It is noteworthy that with an increase in the AFP content, the contact area between ice and asphalt, and hence adhesion, decreases (Figure 9), and during the pull-out test the sample with AFP less than 3% showed a greater anti-icing effect [54].

Thus, the use of hydrophobic and superhydrophobic coatings is very promising in course of the prolongation of the service life of the materials and the reduction of the cost for their repair. However, it has also been reported that hydrophobic and superhydrophobic additives can reduce the mechanical strength of the material [35]. Meanwhile, it is necessary to further study the theory of surface hydrophobicity, wear resistance of hydrophobic materials, improve the existing techniques for their formation and reduce their cost.

## 3. Electrothermal Composite Materials for De-Icing Applications

Various composites with thermoelectric properties have found effective application in everyday life. Among them, special attention is paid to their anti-icing properties, which is important for work in cold climates [59,60]. Such materials can be obtained by introducing electrically conductive components into the structure of insulators and poor electrical conductors. Among these materials are 1D carbon fibers, carbon nanotubes, nanowires [61,62], 2D graphene [63] 3D metal particles [64,65] and other materials.

**Figure 10:**
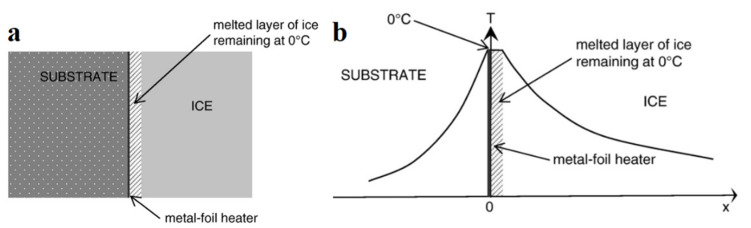
(**a**) Scheme of the anti-icing heating film on the substrate surface; (**b**) the temperature distribution at the interface after heating the film. Reprinted with permission from [66].

To implement this de-icing technology, a simple process diagram has been proposed, according to which a heating material is applied to the substrate surface in the form of a thin film (Figure 10a). When the heating material is turned on, the temperature at the interface between the substrate and ice rises, thereby contributing to the melting of ice (Figure 10b) [66].

**Figure 11:**
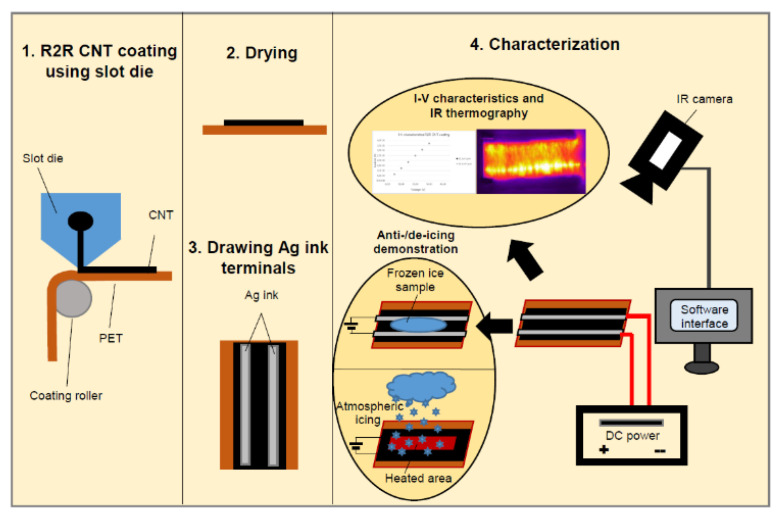
Scheme of obtaining CNT films by the roll-to-roll method and their testing. Reprinted with permission from [79].

Carbon materials due to their unique properties have found application in the anti-icing technology [67,68,69,70,71,72,73,74,75,76,77,78]. Rashid et al. created a heating material in the form of thin films with carbon nanotubes (CNTs) [79]. Using the roll-to-roll production method of coating with a slot die, the CNT suspension is applied to a substrate in the form of a film based on polyester (PET) (Figure 11). According to the authors, it is possible to obtain a continuous coating with a size of a square meter by this method. The test work on de-icing CNT films at subzero ambient temperatures in atmospheric pressure was performed.

**Figure 12:**
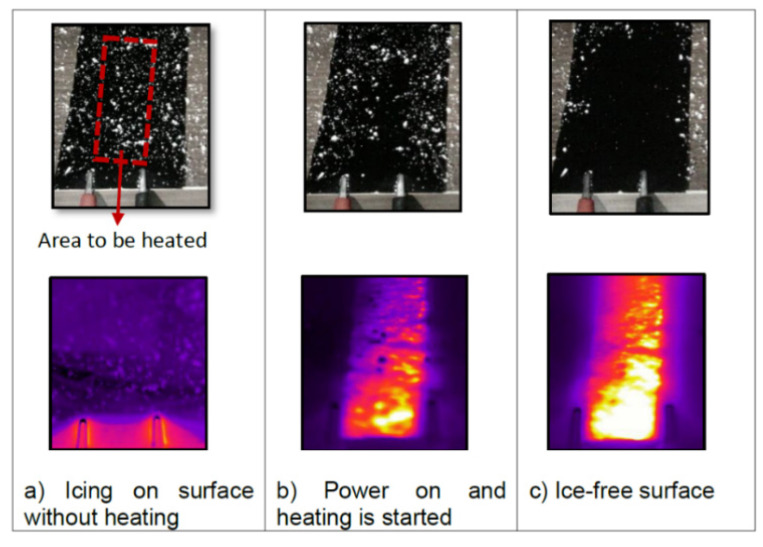
Demonstration of anti-icing effect of the sheet coated by CNTs produced by the roll-to-roll method (IR and color images) under atmospheric icing conditions. Reprinted with permission from [79].

By measuring the electrical and thermal properties using infrared thermography, it was determined that among all the samples, the most effective were the samples with CNT suspensions of 2 wt.% and 3 wt.%. Their electrical resistance values are 806 ohms and 23.2 kΩ, respectively. Figure 12 presents an experiment, in which the ice was removed by using CNT sample of 2 wt.% (Size 25.5 cm^2^) in the open air. After removing the ice, the sample further prevented the formation of ice on its surface.

**Figure 13:**
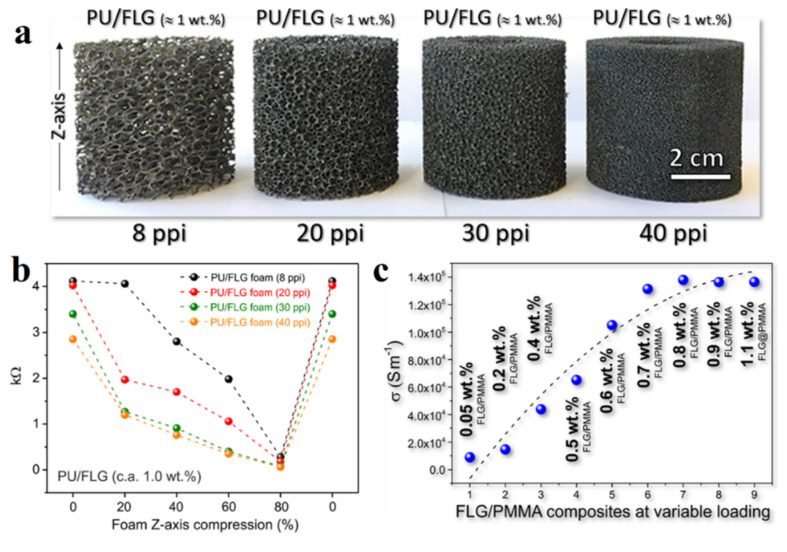
(**a**) Photos of composites FLG/PU ~1 wt.% with different hole values; (**b**) dependence of the FLG/PU sponge resistance on the degree of its deformation; (**c**) the change in the FLG/PMMA electrical conductivity of the composite in dependence with the coating/drying cycles. Reprinted with permission from [76].

On the basis of organic polymers, which are electrical insulators, it is also possible to obtain heating composites [80,81,82]. In [76], a group of scientists created electrically conductive materials based on organic polymers such as polyurethane (PU), acrylonitrile-butadiene-styrene (ABS) and polymethyl methacrylate (PMMA) and polyethylene ether ketone (PEEK) by coating their surfaces with multiple layers of graphene (FLG). Figure 13a presents the cylindrical PU sponges in the form of polymer meshes with different sizes (from 8 to 40 ppi) that were coated with FLG by immersing them into a graphene suspension, and then drying. After several cycles, composites based on FLG/PU with ~1 wt.% were obtained. Obviously, the higher the ppi PU value of the sponge, the more a graphene coating is formed in its structure. It is determined that the resistance of composites is dependent on the degree of their compression along their *Z*-axis (Figure 13b). This is due to the fact that with an increase in the sponge compression, the distance between the conductive PU fibers with the FLG coating becomes closer leading to a decrease in resistance. At the same time, when the sponge is restored to its original shape, its resistance is also restored to the original value. In the case of the FLG/PMMA composite, it was found that the conductivity of the samples also increased with the number of cycles of the coating/drying procedure (Figure 13c).

**Figure 14:**
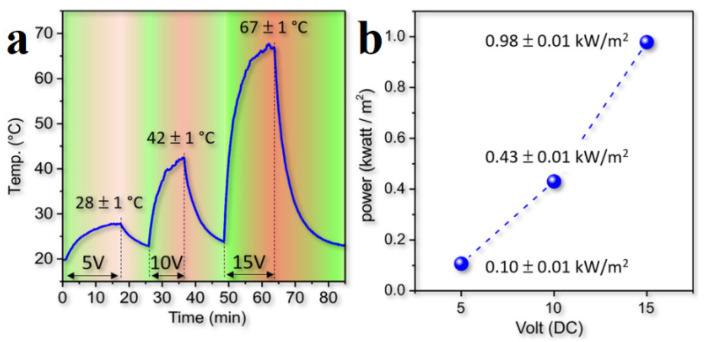
(**a**) Dependence of the temperature value on the applied potential and (**b**) the thermal power density of the composite (kW/m^2^) as a function of the applied external potential for 0.10 wt.% FLG/PMMA composite. Reprinted with permission from [76].

The temperature of the composite surface as a function of time at different values of the potential was also measured. For a 0.10 wt.% FLG/PMMA composite, the surface temperature limit was 67 ± 1 °C at 15 V (Figure 14a). According to the relationship between the density of the thermal power of the composite (kW/m^2^) required to achieve a given temperature value at a given time and the applied external potential (V), this technology can replace traditional electric heaters and radiators (Figure 14b). These coatings based on low FLG concentration are capable of both quickly heating and cooling due to their thin structure.

The formation of ice on the surface of an aircraft poses safety hazards, among which are the decrease in the aircraft’s controllability due to the roughness of the surface, the increase in resistance, leading to an additional load on the engines, and to an increase in fuel consumption. To avoid such incidents, anti-icing fluids have been developed, which are used to treat the surfaces of critical parts of the aircraft. In order to save money, such fluids are diluted with water, and thickeners are also added to them to increase their service life. However, this method is costly. In the case of superhydrophobic coatings, problems arise with their durability in flight conditions. The cover for aircraft should be kept on the surface as strong as possible; in this case, strong wind and vibration of the fuselage in flight will help to get rid of ice.

**Figure 15:**
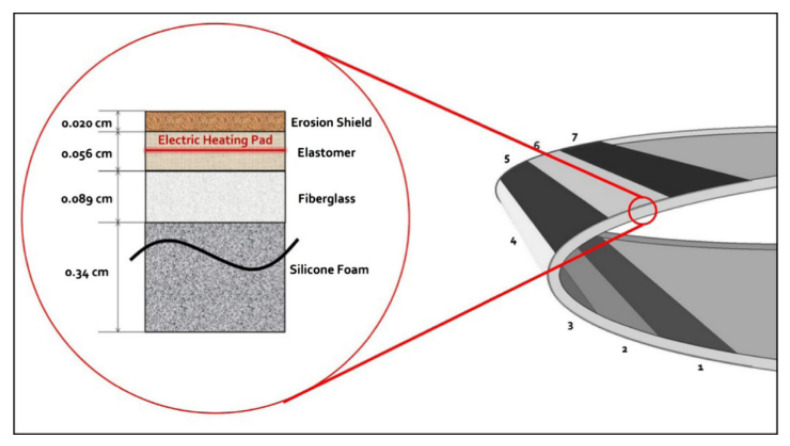
Electro-thermal heating coating built-in four-layer composite panel for de-icing purposes. Reprinted with permission from [83].

In order to avoid icing, there are used systems, which, according to the principle of operation, can be divided into categories: chemical, mechanical and thermal. Among them, the most promising are the electrothermal systems (Figure 15) [83]. Their efficiency is based on the integration of several heating coatings into the aircraft skin, which can be activated simultaneously for both anti-icing and ice removal from the surface.

**Figure 16:**
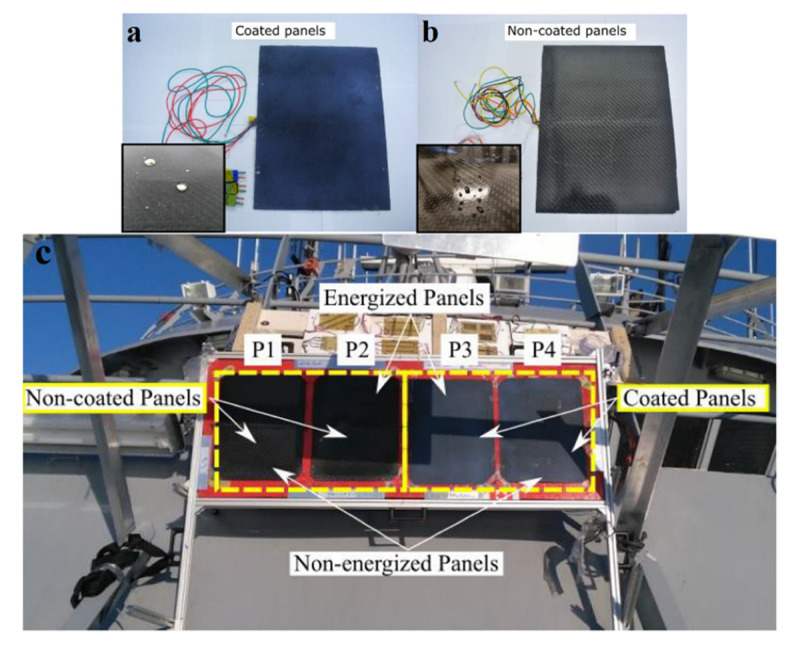
(**a**) Panel with a hydrophobic coating presenting water droplets protruding from the surface; (**b**) an uncoated panel demonstrating that water droplets slightly wet the surface; (**c**) photographs of different panels installed on board the ship. Reprinted with permission from [84].

Previously, 3D printing technologies were limited in the creation of anti-icing composites due to the difficulty of printing continuous conductive fillers in the composite structure. However, some scientists claim that it is possible to obtain a composite of continuous metal wires (heating element) in a polymer matrix on a 3D printer using the fused deposition modeling [84]. As a result, the heating panels with an additional commercial hydrophobic coating (Vernon Hills, IL, USA) were fabricated. To improve the anti-icing properties, they combined a hydrophobic method of de-icing with a thermoelectric one [84,85]. When comparing the photographs of the obtained panels, the effect of surface wettability for a water droplet becomes obvious, since the hydrophobic coating promotes its rolling and removing water droplets from the surface of the panel that are formed as a result of their heating (Figure 16a,b).

To demonstrate the effectiveness of the developed panels, the field tests were carried out onboard the patrol vessel in the Norwegian Sea (Figure 16c). Anti-icing panels with/without hydrophobic coating were compared in the harsh conditions of sea spray, wind and low temperatures (average −6 °C to −20 °C). As a result, it was demonstrated that the additional application of a hydrophobic coating to electrical heating reduces the amount of energy consumed by about 50%, depending on the operating mode.

The formation of ice on the roads often leads to accidents of vehicles, in some cases it ends with the closure of roads until complete clearing of icing. Among the known consequences are cases when people fall when walking on such roads and get serious injuries. To avoid such cases, salt is poured onto the roads, which is harmful to the environment [86]. Less expensive and environmentally friendly methods are now being developed to remove the ice from the surface of concrete, as well as to prevent it from ice forming with the help of electricity [87,88,89,90,91,92,93].

**Figure 17:**
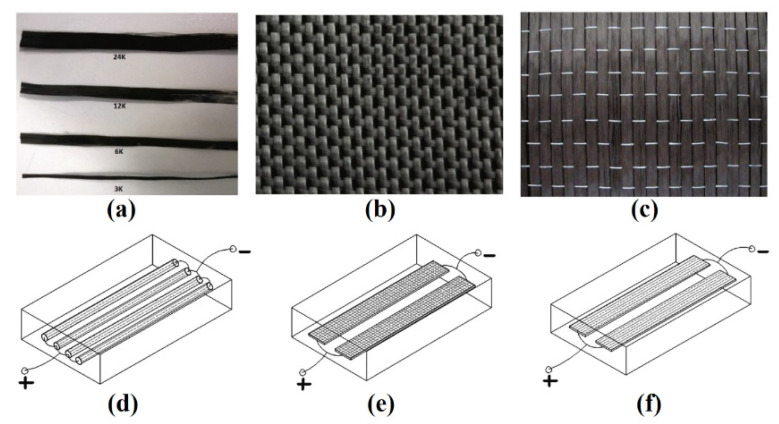
(**a**–**c**) Forms of carbon fibers; (**d**–**f**) schemes of heating panels based on carbon fibers embedded in concrete blocks. Reprinted with permission from [91].

In [91], scientists show a novel method of deicing by electrical heating using three different forms of carbon fibers. Nowadays, four grades of carbon fibers are produced in the world: 3 K, 6 K, 12 K and 24 K (Figure 17a). Their name is associated with the number of single fibers collected in one bundle. When comparing the cost of carbon fibers, woven carbon fibers are more expensive (Figure 17b) than unidirectional fibers (Figure 17c). When measuring the resistance of fibers of 3 K, 6 K, 12 K and 24 K grades with a length of 1 m, their values turned out to be as follows: 68 Ohm, 34 Ohm, 17 Ohm and 8.5 Ohm, respectively. Although the resistance of the individual fibers was constant, due to the complex structures, the resistance for woven and unidirectional fiber fabrics was not constant. As a result, concrete blocks were assembled into which three types of fibers were embedded (Figure 17d–f).

The results of investigations based on comparing the time to reach 0 °C for concrete blocks with different types of carbon fibers embedded are shown in Table 1. The heating characteristics of the carbon filament and unidirectional carbon fabric were found to be similar.

**Figure 18:**
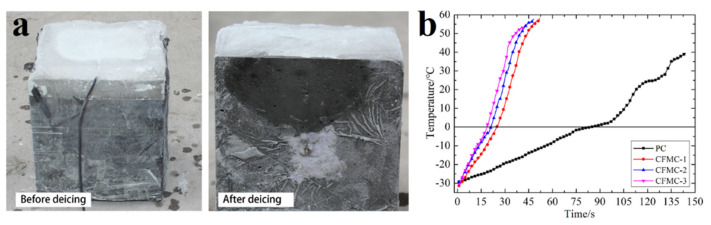
(**a**) Photographs of ice on the concrete surface before and after absorption of microwaves; (**b**) the heating rate of CMFC of different fibers concentrations compared to pure concrete. Reprinted with permission from [101].

Microwave ice removal is a method characterized by rapid and selective heating and higher thermal efficiency [94,95,96,97,98,99,100]. In [101] the effectiveness of removing ice from the surface of concrete with the addition of modified carbon fibers (carbon fiber modified concrete (CMFC)) is discussed. In this case, there is a change in the electromagnetic properties of concrete due to which the absorption of microwaves by the concrete is improved (Figure 18a). Test results show that with increasing carbon fiber concentration, the microwave energy absorption of the CFMC increases in the beginning. When the microwave frequency is around 2.45 GHz, the real part of the complex dielectric constant of CFMC with carbon fiber content of 1‰ (CFMC-1), 3‰ (CFMC-2) and 5‰ (CFMC-3) is 1.59 times, 1.69 times and 0.94 times, higher than that for conventional concrete (PC).

In addition, the results of tests for de-icing with microwaves demonstrate that CFMC-3 has the fastest heating rate of 1680 °C/s, which is 4.46 times higher than that of PC (Figure 18b). Even the presence of only 1‰ of carbon fiber in the structure of a concrete matrix can increase its heating rate up to 3.42 times.

Multiwalled carbon nanotubes (MWCNT) can also be applied for de-icing purposes [85,102,103,104,105,106]. In [107], the creation of a system consisting of a carbon nanofiber polymer (CNFP, 10–200 nm) as a heat source, an AlN-ceramic based insulating layer (0.5 mm), a MWCN/cement thermal conductive layer and a thermally insulated substrate is presented. The MWCNT/cement based composite with 3 wt.% of MWCNTs was utilized as a heat transfer layer, as its thermal conductivity, is significantly higher than that of cement with other fillers and conventional cement based composites.

**Figure 19:**
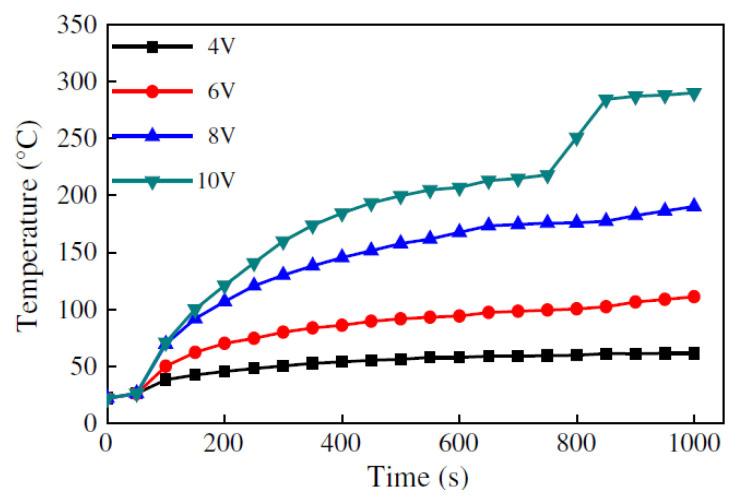
Curves of temperature dependence on different applied voltages at room temperature. Reprinted with permission from [107].

When tested at different voltages (4, 6, 8 and 10 V) at room temperature, it was found that the temperature of the CNFP increased rapidly in a short period of time due to the unique electrothermal properties of the single CNF and the CNFP microrelief (Figure 19).

Despite the good results, there are a number of other problems that hinder the use of anti-icing techniques on a large scale. One of these is the economic disadvantage of using microwave absorbers and conductive fillers in large areas of road surface [108,109,110,111]. In addition, the effectiveness of heating and absorbing microwaves of materials depends on their location in the asphalt/concrete structure. Placing special fillers/heating systems close to the concrete surface has a positive effect on thermal performance, however their placement very close to its surface negatively affects its durability.

For anti-icing systems, their installation is required at the stage of roadway construction, since this affects the volume of operation and the cost of the system. In order to save energy, systems with heating should not work unnecessarily, they must be manually controlled, but it is much more practical to use the automatic mode of turning on and off heating, which will independently work with the help of special sensors only when there is a danger of ice formation. It is also important that after the ice has melted, its additional removal is required, since the accumulated liquid after turning off the system will turn back into ice. To avoid this, it is necessary to take into account the ways to remove the melted snow and ice from their initial place of heating at the design. During liquid, electrothermal heating and heating using radiation, the authors do not take into account the need to establish heat-insulating layers, which will help prevent heat loss due to soil heating.

## 4. Conclusions

This review summarizes the research activity related to the protection of surfaces from icing. The de-icing methods related to the use of hydrophobic coatings, thermoelectric heating, and microwave exposure are briefly described. Despite the effectiveness of removing ice from the surface, for the widespread use of a certain method the following characteristics such as energy saving, reliability in a long period of their operation and environmental safety are important. For example, for hydrophobic coatings, the parameters of their wear resistance and slip resistance are important. In the case of using nanomaterials, despite their unique properties, their addition to the concrete structure may not be economically profitable. Most of the published papers demonstrate the effectiveness of such developments, however they are not widely used in everyday life, leaving the potential for the further developments in this direction.

In the case of using various salts and polymer coatings for anti-icing purposes, the research should be focused on the addition of various agents due to which, the anti-icing, environmental friendliness, adhesion and anti-corrosion properties would be improved. At the same time, such substances should either reduce the adhesion of ice (as much as possible to reduce the force of adhesion of ice to the surface and maintain this ability for a long period of operation), or lower the freezing point of water on the protected surface. In the case of using electricity, besides its cost, the additional supervisory personnel are required to monitor the operation of technical equipment and their safety. In this regard, it is necessary to develop automatic systems for their independent operation.

## Figures and Tables

**Table 1:** Amount of time to reach 0 °C with three different heating panels. Reprinted with permission from [91].

Heating Panel	Time to Reach 0 °C (min)			
	−5 °C	−10 °C	−20 °C	−30 °C
Carbon filament	30.17	66.10	207.50	Not sufficient
Unidirectional carbon fabric	30.25	67.33	208.83	Not sufficient
Woven carbon fabric	39.33	83.50	226.67	Not sufficient
